# In silico identification of variations in microRNAs with a potential impact on dairy traits using whole ruminant genome SNP datasets

**DOI:** 10.1038/s41598-021-98639-9

**Published:** 2021-10-01

**Authors:** Céline Bourdon, Mekki Boussaha, Philippe Bardou, Marie-Pierre Sanchez, Sandrine Le Guillou, Thierry Tribout, Hélène Larroque, Didier Boichard, Rachel Rupp, Fabienne Le Provost, Gwenola Tosser-Klopp

**Affiliations:** 1 Université Paris-Saclay, INRAE, AgroParisTech, GABI, 78350 Jouy-en-Josas, France; 2 Sigenae, INRAE, 31326 Castanet-Tolosan, France; 3 GenPhySE, Université de Toulouse, INRAE, ENVT, 31326 Castanet-Tolosan, France

**Keywords:** Gene regulation, Genetic variation

## Abstract

MicroRNAs are small noncoding RNAs that have important roles in the lactation process and milk biosynthesis. Some polymorphisms have been studied in various livestock species from the perspective of pathology or production traits. To target variants that could be the causal variants of dairy traits, genetic variants of microRNAs expressed in the mammary gland or present in milk and localized in dairy quantitative trait loci (QTLs) were investigated in bovine, caprine, and ovine species. In this study, a total of 59,124 (out of 28 millions), 13,427 (out of 87 millions), and 4761 (out of 38 millions) genetic variants in microRNAs expressed in the mammary gland or present in milk were identified in bovine, caprine, and ovine species, respectively. A total of 4679 of these detected bovine genetic variants are located in dairy QTLs. In caprine species, 127 genetic variants are localized in dairy QTLs. In ovine species, no genetic variant was identified in dairy QTLs. This study leads to the detection of microRNA genetic variants of interest in the context of dairy production, taking advantage of whole genome data to identify microRNA genetic variants expressed in the mammary gland and localized in dairy QTLs.

## Introduction

MicroRNAs are small noncoding RNAs of approximately 22 nucleotides in length that are highly conserved between species^1^. They are involved in posttranscriptional gene regulation through their impact on messenger RNAs (mRNAs). This interaction will lead to the repression of the translation process or to the degradation of the targeted mRNAs, depending on the base-pair binding between the microRNA and the mRNA in the recognition site, the seed sequence^2^. MicroRNAs have important roles in mammary gland development, lactation and milk biosynthesis^3–5^. Few data on miRNA role on milk-producing traits have been described: for example microRNAs like *miR-21* or *miR-143* are abundantly expressed in bovine mammary gland^6^; the *miR-24, miR-145, miR-103, miR-152, miR-224* are involved in milk fat traits in bovine or caprine species^7–10^. Mammary miRNome data, listing the microRNAs expressed in the mammary gland or present in milk, are available in bovine and caprine species^11,12^. In the Holstein breed, the mammary gland miRNomes were described in cows with mastitis^13^ and healthy cows^11^. The miRNomes of whole milk in Holstein and Normande breeds^14^, milk fat in Holstein breeds^15^ and milk fat, whey and somatic cells in Holstein breeds^16^ are also available. In caprine species, the mammary gland miRNome is available from lactating Alpine goats^12^. In ovine species, however, no mammary gland data were available until recently, with the publication of a miRNome by Wang et al*.*^17^. Few data on miRNA role on milk-producing traits have been described: for example microRNAs like *miR-21* or *miR-143* are abundantly expressed in bovine mammary gland^6^; the *miR-24, miR-145, miR-103, miR-152, miR-224* are involved in milk fat traits in bovine or caprine species^7–10^.

Both small genetic variations (SNPs and InDels) and structural variations (SVs) may alter microRNAs via their biogenesis or their role on mRNA expression regulation. In our present work, we only investigated the effect of SNPs and InDels. Indeed, if the genetic variant is located within the seed region, it may affect the binding between the microRNA and targeted mRNAs and thus lead to the nonrecognition of targeted mRNAs or, in contrast, to the recognition of novel mRNAs or novel targets^18^. If the genetic variant is located in another part of the microRNA or in its flanking regions, the microRNA expression level may be impacted^19^.

MicroRNA polymorphisms have been studied in various livestock species, such as chicken, porcine, and bovine, from the perspective of pathology or production traits. Wu et al*.* found a genetic variant in *pri-miR-26a-5p*, the primary transcript of *miR-26a-*5p, inducing a modification of the secondary structure of the microRNA gene^20^. The abundance of the mature microRNA is decreased when the mutation is present, impacting chicken egg production traits^20^. Similarly, a SNP in porcine *miR-208b* was predicted to affect the secondary structure of *pri-miR-208b* and therefore the expression of the microRNA precursor *pre-miR-208b*, the mature microRNA, and the expression of the targets *SOX-6* (SRY-Box Transcription Factor 6) and *MYH7* (Myosin heavy chain 7) genes*.* Differences in mRNA levels affect the density of muscular fibers and thus muscle and meat quality traits^21^.

In bovine species, a genetic variant in the seed region of *bta-miR-2899* was detected (chr18: 42,198,087 G > A), localized in a QTL affecting the somatic cell score (SCS), and associated with the presence of mastitis. An impact on the targeted mRNA *SPI1* (Spi-1 proto-oncogene), which is a potential candidate factor for inflammation in the bovine, was also investigated. The *SPI1* relative expression in mammary glands of cows with *bta-miR-2899* with the AA genotype (low SCS) was significantly higher than those with the GA or GG genotype (high SCS)^22^.

Bovine, caprine and ovine ruminants provide 86% of the world milk production^23^. In these three species, the improvement of dairy traits has always been a core objective of breeding programs and remains in the era of genomic selection. In genomic evaluation, breeding values are usually predicted using SNPs spread across the genome, that capture the causal variant effects, thanks to linkage disequilibrium. Although using sets of neutral markers for genomic evaluation is efficient, adding causal variants could improve the reliability of the estimated genomic breeding values, as mentioned by Oget et al., who compared different methods^24^. Numerous studies conducted in all three species described genomic regions or quantitative trait loci (QTLs) associated with dairy traits. Some of these QTLs are currently available in the public AnimalQTLdb database^25^, and additional studies have listed QTLs for milk production, milk composition, and mastitis resistance traits (see, for example^24,26–28^). However, the identification of causal mutations is still challenging.

Missense mutations located in the *DGAT1* (Diacylglycerol O-acyltransferase 1) and *PAEP* (Progestagen-associated endometrial protein) genes were known to be causative for milk production and composition^27,29–31^. *DGAT1* encodes an enzyme involved in fatty acid metabolism in milk, while *PAEP* encodes β-lactoglobulin, which is the most abundant whey protein in bovine milk. In addition to these two examples, causal variants located in coding regions of genes are scarce, and most of the candidate variants that are proposed as causative for quantitative traits, and in particular dairy traits, are located in noncoding regions. Indeed, noncoding regions represent approximately 98% of mammalian genomes and have functional impacts on biological processes^32–34^. In the GWAS (genome-wide association studies), approximately 90% of the candidate variants are localized within these noncoding regions which can have regulatory roles^35,36^. Transcription start sites, enhancers or promoters of genes are noncoding regions leading to the activation of genes in different tissues^37–40^. Noncoding RNAs, which are not translated into proteins, could modulate gene expression and impact biological systems, such as milk production or development of the mammary gland^41,42^.

Some microRNA variants still remain unexplored. The aim of this study was to detect putative causal microRNA variants that could influence dairy traits in three ruminant species. To target microRNA variants that could potentially be the causal variants for these traits, only genetic variants of microRNAs expressed in the mammary gland or present in milk and localized in dairy QTLs were investigated. To this end, we developed a pipeline to filter microRNA genetic variants from whole-genome variants in bovine, caprine and ovine species.

## Results

### Detection of dairy microRNA genetic variants

Out of a total of 28 million genetic variants in bovine, 87 million in caprine, and 38 million variants in ovine species, 138,442, 26,777 and 4769 microRNA variants were identified, respectively.

Of them, only the variants located in the microRNA genes of the dairy miRNomes were retained, i.e., 895 bovine, 239 caprine and 906 ovine microRNAs. After this filter, 59,843, 13,427, and 4761 variants were kept in bovine, caprine, and ovine species, respectively.

Finally, in bovine, the selection of genetic variants with a frequency higher than 0.01 led to a total of 59,124 genetic variants (SNPs and small InDels) of microRNAs expressed in the mammary gland or present in milk. Among them, 360 genetic variants were located in a microRNA precursor, with 213 microRNAs presenting at least one variant. Of them, 101 variants were located in 75 mature microRNAs, including 44 in a seed region of 37 different microRNAs (Table 1). The 58,764 additional genetic variants were located in flanking regions of microRNAs. In caprine species, 13,427 microRNA genetic variants were filtered, with 100 microRNAs presenting at least one variant. Of them, 33 variants were located in a precursor of 24 different microRNAs, including four variants in three mature microRNAs and one genetic variant in a seed region of *chi-miR-425*. The script with ovine data resulted in 4761 microRNA genetic variants in 106 different microRNAs. Of them, 33 variants were located in the precursor of 19 microRNAs, with six of them in five mature microRNAs and one variant in the seed region of *oar-miR-539* (Table 1). In addition, the microRNA *miR-539* presented a genetic variant in bovine species in a 100 bp-flanking region of this microRNA.

**Table 1:** Number of microRNA genetic variants detected, with and without the dairy QTL filter, for the three different species.

	Without QTL data				Dairy QTL			
	Total	Precursor	Mature	Seed	Total	Precursor	Mature	Seed
Bovine	59,124	360	101	44	4679	26	10	3
Caprine	13,427	33	4	1	127	0	0	0
Ovine	4761	33	6	1	0	0	0	0

**Figure 1:**
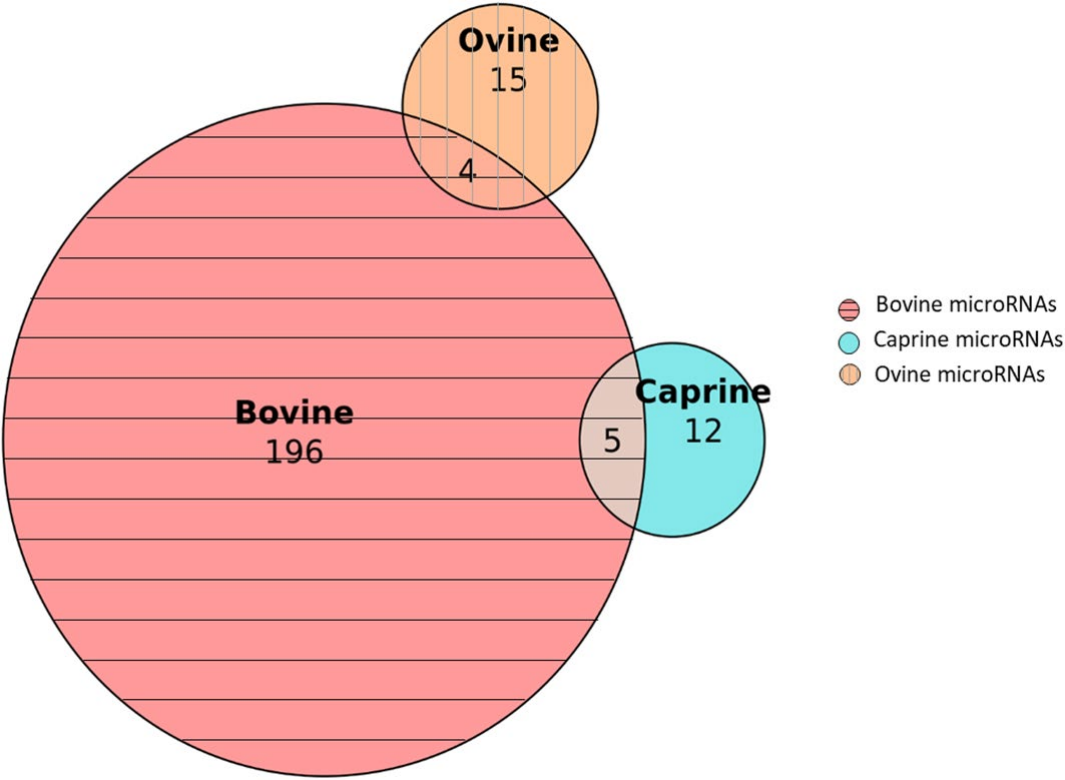
Number of microRNAs presenting at least one variant in a precursor in bovine, caprine and ovine species. Five microRNAs present variants both in bovine and caprine species. Four microRNAs present variants both in bovine and ovine species.

**Figure 2:**
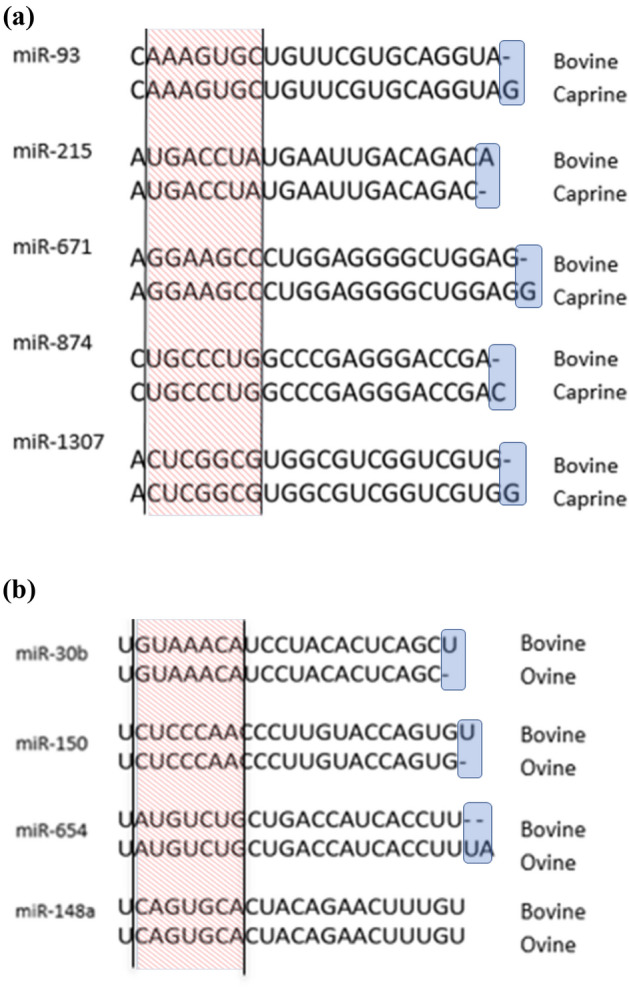
(**a**) Nucleotidic sequence of the five microRNAs presenting genetic variants in the precursor of both bovine and caprine species. The differences are located at the end of each microRNA. (**b**) Nucleotidic sequence of the three microRNAs presenting genetic variants in the precursor of both bovine and ovine species. The seed regions of the microRNAs are indicated between the black traits, in the red stripes. The nucleotidic changes are highlighted in blue squares.

In these analyses, five microRNAs (*miR-93, miR-215, miR-671, miR-874,* and *miR-1307)* had variants in their precursor in both bovine and caprine species (Fig. 1). These five microRNAs are very similar in the two species, with the same seeds and, therefore, the same predicted mRNA targets. They differ by only one nucleotide at the 3′ end of the mature sequence (Fig. 2a).

Four microRNAs, *miR-30b, miR-148a, miR-150,* and *miR-654,* had variants in both bovine and ovine species (Fig. 1). The microRNAs had very similar sequences with identical seeds and therefore the same predicted mRNA targets (Fig. 2b).

Out of the 28 million bovine genetic variants (SNPs and InDels), 59,124 were variants of microRNAs expressed in the mammary gland or present in milk, of which 4679 were located in dairy QTLs. Of these, 1,044, 573, 140, and 69 variants were located within 1 kbp, 500 bp, 100 bp, and 50 bp flanking regions of 80, 77, 56, and 38 different microRNAs, respectively (Table 2, Supplementary Table S1). Twenty-six variants, located in 21 different microRNA precursors, were found in QTL regions associated with six different dairy traits: protein content, protein yield, fat content, fat yield, somatic cell count, and milk yield. These 26 genetic variants corresponded to 22 SNPs, two insertions (one and two nucleotides) and two single nucleotide deletions (Table 3). Eight genetic variants (6 SNPs, 1 insertion and 1 deletion) were identified in seven different mature microRNAs in QTL associated with five traits: protein content, protein yield, fat content, fat yield, and somatic cell count. Finally, three SNPs were present in the seed regions of three different microRNAs, *bta-let-7e*, *bta-miR-2888* and *bta-miR-449b*, in QTL regions associated with protein content, protein yield, and fat yield. Out of the 26 genetic variants located in microRNA precursors, 20 were found in Holstein, four in Normande, and one in Montbéliarde breed, and one genetic variant was found in both the Holstein and Montbéliarde breeds. The frequency of the variants as well as their positions within CpG islands were also considered in this study (Table 3). A total of 74 microRNA genetic variants, including four microRNA precursors, are located in CpG islands. None of the microRNA variants located in QTL regions were located in TFBS.

**Table 2:** Number of detected bovine microRNA genetic variants of interest, according to their locations.

Variant location	Number of variants/number of different microRNAs	Variants with frequency ≥ 10%/number of different microRNAs	Number of variants in CpG islands/number of different microRNAs
Seed region	3/3	0	0
Mature microRNA	8/7	1/1	0
microRNA precursor	26/21	8/8	4/4
± 50 bp	69/38	33/20	17/8
± 100 bp	140/56	66/33	27/10
± 500 bp	573/77	291/61	50/14
± 1000 bp	1044/80	597/78	74/24

The number of microRNAs presenting at least 1 variant was mentioned, as well as the number of variants with a frequency ≥ 0.10 and those located in CpG islands.

**Table 3:** Description of the 26 genetic variants detected in the precursor of microRNAs in bovine QTL regions.

Chr	Position	Reference allele	Alternative allele	Breed	Frequency alternative allele	microRNA	QTL trait	Localisation
3	102923794	G	A	Holstein	0.136	*bta-miR-2415*	Milk yield and quantity of proteins	Precursor
4	114614954	T	G	Holstein	0.047	*bta-miR-671*	Milk yield and fat content	Precursor
5	30251690	G	C	Holstein	0.673	*bta-miR-2425*	Protein yield	Precursor
5	30952192	T	G	Holstein	0.038	*bta-miR-2426*	Protein yield	Precursor
5	62117780	G	A	Holstein	0.019	*bta-miR-135a-2*	Fat content	Precursor
5	84244517	G	A	Holstein and Montbéliarde	0.071 and 0.017	*bta-miR-2436*	Fat content and fat yield	Precursor
5	101542291	C	T	Holstein	0.019	*bta-miR-2284r*	Fat yield	Precursor
5	118347364	C	CT	Holstein	0.012	*bta-miR-2284h*	Protein yield	Precursor
6	99976608	T	C	Normande	0.048	*bta-miR-2446*	Protein yield	Mature
6	99976613	G	A	Holstein	0.013	*bta-miR-2446*	Protein yield	Precursor
7	50687460	T	C	Holstein	0.210	*bta-miR-874*	Fat content	Precursor
7	63886927	C	CCA	Holstein	0.026	*bta-miR-2461*	Quantity of proteins and fat content	Mature
15	34628957	C	T	Holstein	0.296	*bta-miR-2313*	Fat content	Precursor
18	56407853	TC	T	Holstein	0.056	*bta-miR-150*	Quantity of proteins	Precursor
18	56407916	T	G	Holstein	0.013	*bta-miR-150*	Quantity of proteins	Mature
18	58015050	G	A	Holstein	0.051	*bta-let-7e*	Quantity of proteins	Seed
18	61145895	G	T	Normande	0.012	*bta-miR-371*	Protein yield	Precursor
19	38542897	CG	C	Holstein	0.304	*bta-miR-2886*	Protein yield and somatic cell count	Mature
19	39081170	T	C	Holstein	0.903	*bta-miR-152*	Somatic cell count	Precursor
20	23967291	G	T	Normande	0.083	*bta-miR-449b*	Fat yield	Seed
20	23967292	T	A	Normande	0.083	*bta-miR-449b*	Fat yield	Mature
21	36134549	C	T	Montbéliarde	0.121	*bta-miR-2888-1*	Protein yield	Precursor
21	36134560	T	G	Montbéliarde	0.044	*bta-miR-2888-1*	Protein yield	Mature
25	35300154	G	C	Holstein	0.051	*bta-miR-2388*	Protein yield	Precursor
25	35300168	G	A	Holstein	0.756	*bta-miR-2388*	Protein yield	Precursor
29	45520815	C	T	Holstein	0.294	*bta-miR-2408*	Protein yield	Precursor

The same approach led to a total of 127 genetic variants in caprine species. These variants impacted only one microRNA, *chi-miR-22*. All of those variants were located upstream and downstream of the precursor microRNA, and the nearest was located 14 nucleotides apart from the precursor. These genetic variants were located in a dairy QTL associated with fat yield. They were identified in 73 caprine breeds, including the Alpine and Saanen dairy breeds. No variant was found in the microRNA *miR-22* in bovine species.

In ovine species, no genetic variant was identified using this bioinformatic script.

**Figure 3:**
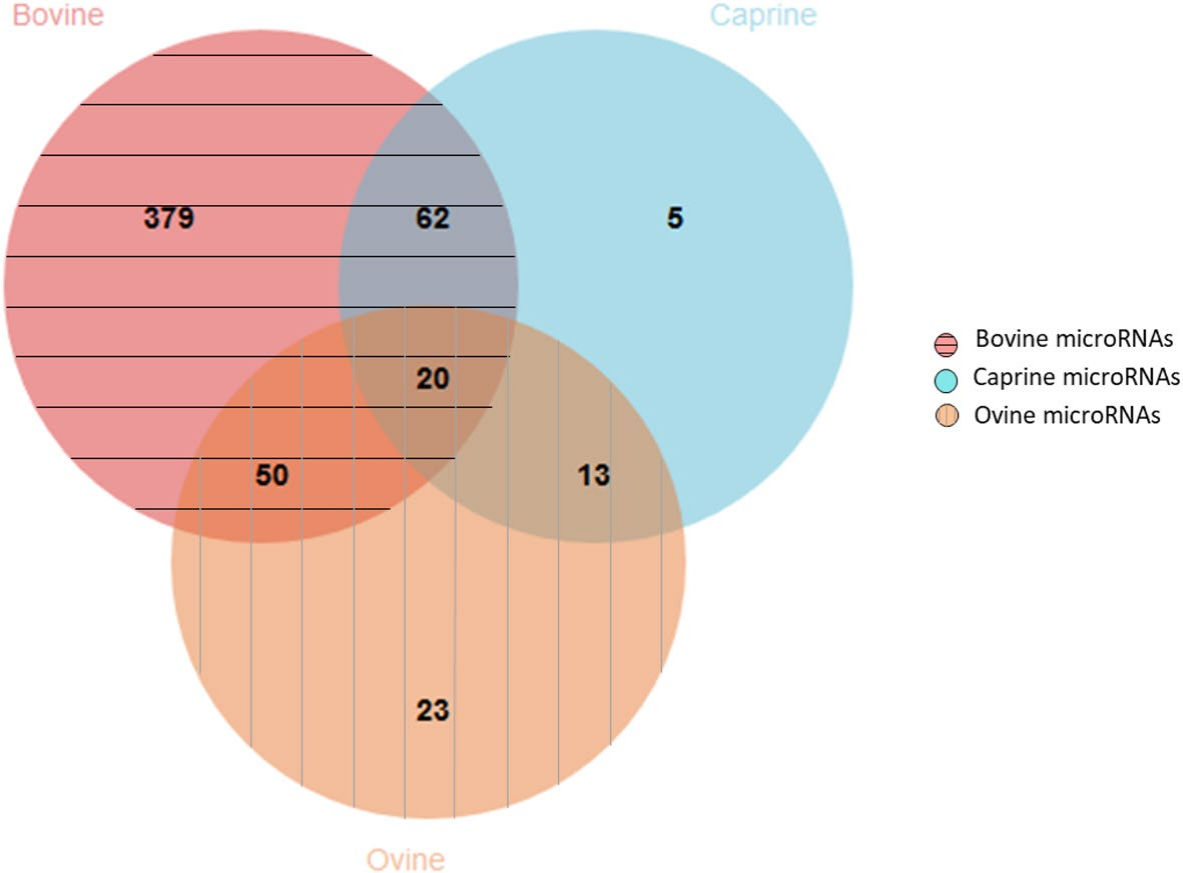
Number of microRNAs presenting at least one variant in bovine, caprine or ovine species and common variants.

In the three dairy species, the analysis led to 511, 100 and 106 microRNAs showing at least one variant in bovine, caprine, and ovine species, respectively, without considering colocalization with dairy and mastitis QTLs (Supplementary Table S1). More specifically, five microRNAs had variants only in caprine species, while 23 microRNAs had variants only in ovine species. Finally, 20 microRNAs had genetic variants in all three ruminant species (Fig. 3).

### Prediction of putative mRNA targets

To find a functional link between microRNAs impacted by the genetic variants detected (Table 1) and dairy traits, the putative mRNA targets of microRNAs were predicted using in silico analyses.

*miR-539*, showing a variant in its seed sequence in the ovine, has 5,300 putative target mRNAs, including 63 and 31 mRNAs that are up- and down-regulated, respectively, in the mammary gland during lactation *versus* non-lactation periods. The *miR-425* with a variant in its seed sequence in caprine species is predicted to have 1,621 putative target mRNAs, 25 of which were found to be differentially expressed by Dai et al*.*^43^ in the mammary gland during lactation *versus* non-lactation periods (5 were down-regulated and 20 were upregulated).

Among the 305 transcripts that are predicted as putative targets of the microRNA *miR-874*, with genetic variants in both bovine and caprine microRNA precursors, four putative targets of *miR-874* are shown to be differentially expressed between lactation and non-lactation periods^43^. *ESRRA* (estrogen-related receptor alpha) and *ATF3* (activating transcription factor 3) are upregulated during the lactation period compared to the non-lactation period, while *GREB1* (growth regulation by estrogen in breast cancer 1) and *CD248* (CD248 molecule) are down-regulated^43^.

For the microRNA *miR-1307,* which is polymorphic in both bovine and caprine species, 11 putative targets were found in the bovine mammary gland transcriptome data. Ten of them were reported to be upregulated (*ADAM12* ADAM metallopeptidase domain 12, *AGPAT6* 1-acylglycerol-3-phosphate acyltransferases 6*, FASN* fatty acid synthase*, MT1A* metallothionein-1A*, PMM2* phosphomannomutase 2*, PTPRT* protein tyrosine phosphatase receptor type T*, RND1* Rho family GTPase 1*, SLC2A4* solute carrier family 2 member 4*, SULF2* sulfatase 2*,* and *THBS2* thrombospondin 2), and one of them was found to be downregulated (*PRR15L* proline rich 15 like), during lactation *versus* non-lactation periods^43^.

### QTL information depending on species

On average, 21.8, 4.6, and 1.7 variants per kbp were detected in bovine, caprine, and ovine species, respectively.

The addition of the “dairy and mastitis QTL” filter led to a large decrease in the number of genetic variants. To compare the data, differences in QTL information available in the three species were studied.

**Figure 4:**
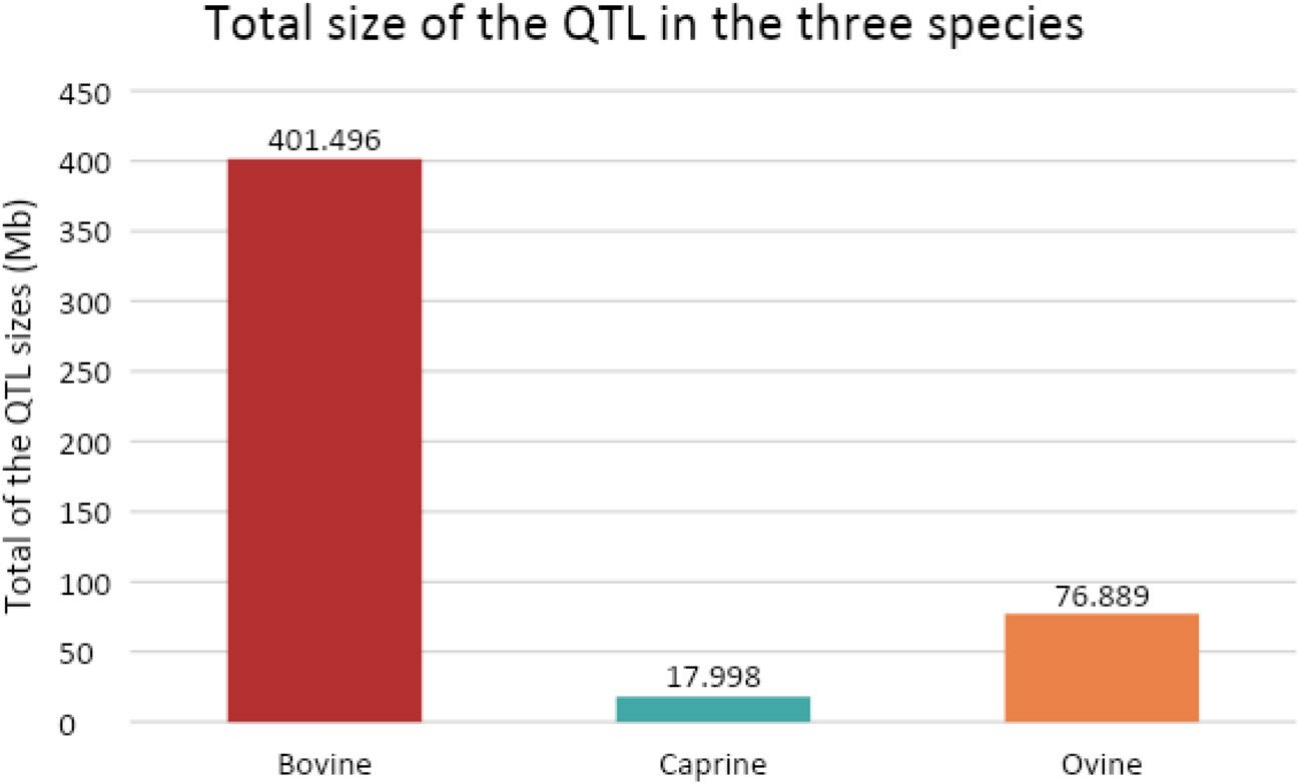
Total size of the whole QTL regions depending on the species, in megabases (Mb).

The number of QTLs differed depending on the species. In the bovine, caprine and ovine species, the QTLs cover 401.5, 18.0, and 76.9 Mbp of the genome, respectively. The mean size of the QTLs, between the bounds of the confidence interval, varies between species: 815 kb in bovine, with QTL sizes from 190 to 4,156,369 bp, approximately 226 kb in caprine, with a range of 174,986 to 403,132 bp, and approximately 1234 kb in ovine species, from 766,403 to 1,997,460 bp. Therefore, the number of QTLs and the length of the genome covered by these regions were much higher in bovine than in the two other species. The total size of the QTLs in bovine is more than fivefold longer than that observed in ovine and more than twenty-two fold longer than that in caprine species (Fig. 4). In bovine QTLs, the number of variants in microRNA genes reached 11.65 per megabase, and 7.06 caprine QTLs were identified.

## Discussion

MicroRNA genetic variants are well studied in humans to diagnose or provide a prognosis for diseases such as cancer or cardiovascular disease^44–47^. MicroRNA databases, in particular the MSDD (MicroRNA SNP Disease Database)^48^ and the miRSNP database^49^, have been developed in this context. Other databases, such as EpimiRBase or miRCancer, provide access to the microRNAs linked with human databases^50,51^.

Approximately 10% of human microRNA precursors contain SNPs, with less than 1% located in seed regions^52^.

In the present study, microRNA genetic variants were studied in the context of dairy traits and in three different dairy ruminant species: bovine, caprine, and ovine species.

A total of 59,124, 13,427, and 4761 genetic variants in microRNAs expressed in the mammary gland or present in the milk were identified in bovine, caprine, and ovine species, respectively.

Although the number of whole-genome variants investigated in this study was higher in caprine (87 million) and ovine (38 million) species than in bovine (28 million) species, a much higher number of variants in microRNA expressed in the mammary gland or present in milk was observed in bovine species than in the two other species. These differences in genetic variant amounts might be due to the amount of data existing in the different criteria. Knowledge of the different genomes, the number of genetic variants in the different species, and the number of microRNAs described in dairy miRNomes are different depending on the species.

In this study, the microRNAs listed in mammary miRNomes were used. A total of 895 bovine microRNAs, including 701 microRNAs detected in mammary gland^11,13^, 239 caprine microRNAs and 106 ovine microRNAs were then retained. These differences likely explain the larger number of microRNA genetic variants in bovine than in the two other species.

As an example, a total of 59,124 microRNA genetic variants were detected in bovine species. Research on genetic variants of interest in bovine species using caprine miRNome data instead of bovine data led to 18,184 microRNA genetic variants. This is in the same order as the caprine results, which leads to 13,427 caprine microRNA genetic variants. The mammary miRNomes seem therefore to be an important factor explaining the difference in the number of genetic variants of interest in the three studied species. The lack of data regarding the caprine and ovine mammary data may explain the difference in results between species.

Of the variants found in microRNAs expressed in the mammary gland and present in milk, 4679; 127; and 0 genetic variants were identified in dairy QTLs in bovine, caprine and ovine species, respectively. In 2019, Jiang et al*.* have studied genetic variants of microRNA located in mastitis QTL regions in bovine species: 2912 SNPs of interest were detected in 691 microRNAs precursors^22^. In our study, the amount of genetic variants of interest is not as big, since only 26 out of 4679 are located in microRNA precursors. This difference may be due to the size and number of the QTL regions chosen by Jiang et al*.*: 8 health and mastitis QTL traits are chosen. Moreover, no miRNome data are used in their study, inducing a greater number of studied microRNAs and thus more genetic variants of interest^22^. No similar study has been published in caprine nor ovine species.

The detection of genetic variants of interest led to an amount of results more important in bovine species than in caprine or ovine ones. These differential results could be due to the quality of the genome annotations that differ between species. It may also be a result of the difference existing inter-species: the quantity and specificity of the available data. As an example of species specificity of genomes, in ovine species more than half microRNAs are located in only one chromosome (chromosome 18)^53^. Moreover, the available data are not as numerous and as precise in the three studied species: the miRNome data are not accessible in ovine species, and, at a lower level, the QTL traits are, for some of them, different. In addition, the total size of the dairy QTL was longer in bovine species than in ovine (fivefold shorter) and caprine (22-fold shorter) species. Thus, the total genome length in which the microRNA genetic variants were searched was longer in bovine species, which could explain a part of the differences we observed in the three species: the number of variants detected per QTL megabase is 1.65 higher in bovine than in caprine species. This difference in the size of dairy QTLs may also be induced by the difference in criteria used to define the QTLs.

More precisely, the difference in the number of variants in the three dairy ruminant species is highlighted by the mean number of genetic variants of interest (genetic variants of microRNAs expressed in the mammary gland or present in milk) per kilobase: this number is more than fourfold higher in bovine species (21.8 variants per kb) than in caprine species (4.6 variants per kb) and more than 12-fold higher than in ovine species (1.8 variants per kb).

Some of the microRNAs found to contain at least one genetic variant of interest appear to have links with dairy traits. These links can appear through some of their putative mRNA targets, which are genes involved in dairy trait determinism, or through the literature, describing some of them as actors in milk traits. *AGPAT6*, impacting milk composition^54^, or the leptin *LEP* genes, impacting milk production^55,56^, are putative targets of microRNAs such as *bta-miR-2888*, *miR-1307* and *miR-425*. Another putative target, *PRLR*, has an effect on milk fatty acid composition^57^.

Some microRNAs with genetic variants identified in this analysis, such as *miR-143 and miR-25* in ovine *or miR-150* in both bovine and ovine species, were found to be involved in milk fatty acid and protein metabolism. For example, the microRNAs *miR-143* and *miR-25* were found to be involved in the regulation of milk fat synthesis^58,59^, while *miR-150* was associated with the lactation process and milk protein production^60^.

Among the five microRNAs presenting a genetic variant in their precursor, some are involved in immune responses or dairy traits. For instance, *miR-93* expression is higher in bovine colostrum than in mature milk^61^, and *miR-874* is upregulated between the galactopoiesis and involution lactation stages in Canadian Holstein cows^15,62^.

The microRNAs *miR-30b* and *miR-150* have variants in both bovine and ovine species. Both were previously associated with lactation: overexpression of *miR-30b* caused a lactation defect in mice^4^, and the expression of *miR-150* resulted in the suppression of mRNAs involved in lipid synthesis during secretion^60^.

Interestingly, twenty microRNAs have genetic variants in all three ruminant species: *miR-17*, *miR-25*, *miR-103*, *miR-107*, *miR-136*, *miR-143*, *miR-150*, *miR-191*, *miR-194*, *miR-218, miR-379*, *miR-381*, *miR-410*, *miR-432*, *miR-494*, *miR-495*, *miR-544*, *miR-655*, *miR-758* and *miR-1185*. Among them, 6 microRNAs are impacted by genetic variants in microRNA precursors. The miR-150 is impacted by at least one genetic variant of interest in its precursor in bovine and in ovine species (in ovine species genetic variants are located in flanking regions). The miR-218 is impacted by at least one genetic variant of interest in its precursor in bovine and caprine species. The *miR-191*, *miR-194*, and *miR-544* show also at least one genetic variant of interest in their precursor in bovine species. In ovine, the *miR-495* also presents at least one variant in precursor.

Some of these microRNAs in common could be of interest in a dairy context. For example, the *miR-103* and *miR-107* microRNAs contain genetic variants in all three species studied and belong to the same microRNA family, thus show identical seed sequences in bovine, caprine and ovine species^63^. These two microRNAs are generally involved in cellular (neuronal) migration^64^, and the chi-miR-103 is also described as mediator of milk fat accumulation in Xinong Saanen dairy goats^9^. The causal role of these variants on dairy traits remains to be demonstrated, but they constitute potential candidate variants for some of the dairy QTLs identified in ruminants. Variants we highlighted in this study in microRNAs could therefore be added to genomic prediction models and participate in the improvement of genomic selection.

## Conclusions

This study led to the detection of microRNA genetic variants of interest in the context of dairy production, taking advantage of whole genome sequence data to identify microRNA genetic variants expressed in the mammary gland and localized in some dairy QTLs. A perspective of this study is to choose some genetic variants and characterize them to test their potential functional impacts.

## Material and methods

### In silico detection of genetic variants

#### VCF files

Similar processes were used for the three bovine, caprine and ovine species. The genomic variation SNPs and small InDels, defined as insertions or deletions shorter than 60 bp located in microRNA genes, were identified using three INRAE databases (all the available data were from the three INRAE databases used), as described in Table 4^65,66^.

**Table 4:** Information on bovine, caprine and ovine genetic variants files.

	Number of variants	Number of animals	Number of breeds
Bovine	28 million	351	16
Caprine	87 million	1124 (+35)	102 (+8 other capra species)
Ovine	38 million	87	12 + 1 crossbreed

Small genomic variations located in bovine microRNA genes were identified using a bovine database containing approximately 28 million SNPs and InDels using whole-genome sequence data from 396 animals corresponding to 18 different breeds (Supplementary Table S2)^65^. Annotation of this database was performed using Variant Effect Predictor v81 (VEP)^67^.

For caprine species, the microRNA small genomic variations were identified using VCF files containing approximately 87 million SNPs and InDels produced from whole genome sequences for 1159 animals, corresponding to 102 *Capra hircus* breeds as well as 8 other capra species for 35 of the animals: *Capra aegagrus, Capra caucasica, Capra pyerenaica, Capra falconeri, Capra ibex, Capra nubiana,* and *Capra siberica.* These data are accessible thanks to the VarGoats project (last update: 1159 goats, November 7th, 2019), corresponding to a resequencing program of 1,000 goat genomes (Supplementary Table S3)^66,68^.

For ovine species, the VCF files used contained approximately 38 million SNPs from 87 animals representing 12 different breeds and 1 crossbreed (Supplementary Table S4).

Briefly, sequence alignments against the UMD3.1 bovine reference genome^69^ which was the latest and widely-used version of the bovine genome when this work started, were carried out using the Burrows-Wheeler Alignment tool (BWA-v0.6.1-r104)^70^ with the “aln” option. The NCBI Capra hircus ARS1 assembly release 102 was used for the caprine species, and Oar_v4.0 version was used for the ovine species. The removal of potential PCR duplicates was performed using the MarkDuplicates tools from the Picard package version 1.4.0 (Broad Institute, http://picard.sourceforge.net). Only properly paired reads with a mapping quality of at least 30 (− q = 30) were retained. The resulting BAM files were subsequently used to the variant calling, using the Genome Analysis Tool Kit 2.4–9 (GATK) version and GATK-UnifiedGenotyper^71^. All the identified variants were then annotated using the Ensembl Variant Effect Predictor (VEP) v81 tool^67^ based on the Ensembl version 81 transcript set and using dbSNP build 143. The SnpEff v4.3 tool permits variant annotation and prediction of their effects in caprine and ovine species^72^.

In caprine species, the process differed in the calling variants step: the SAMtools tool version 1.6 was used at this step as well as the GATK version 3.6 tool^71^. BCFtools version 1.6 and Freebayes version 1.1.0 were also used^73^.

### BED files

#### QTL

In the three species, the QTLs detected for each trait in French breeds are given in Table 5. For each QTL, the genomic location, the nature of trait affected, and the breed(s) in which it was found were specified in the BED file.

**Table 5:** Dairy traits affected by the quantitative trait loci, with the breed for bovine, caprine, and ovine species.

Trait		Bovine			Caprine		Ovine	
		Holstein	Normande	Montbéliarde	Alpine	Saanen	Lacaune	Manech
α-lactalbumin content	a-LA	X	X	X				
β-lactoglobulin content	b-LG	X	X	X			X	
αs1-casein content	as1-CN	X	X	X			X	X
αs2-casein content	as2-CN	X	X	X			X	X
β-casein content	b-CN	X	X	X			X	X
κ-casein content	k-CN	X	X	X			X	
Total caseins	s-CN	X	X	X			X	X
Milk yield	MILK	X	X	X	X	X	X	
Fat yield	FAT	X	X	X	X	X	X	
Protein yield	PROT	X	X	X	X	X	X	
Fat content	FC	X	X	X	X	X	X	
Protein content	PC	X	X	X	X	X	X	
Somatic cell score	SCS	X	X	X	X	X	X	
Clinical mastitis	CM	X	X	X				
Monounsaturated fatty acids	MUFA						X	X
Polyunsaturated fatty acids	PUFA						X	
Unsaturated fatty acids	UFA						X	X
Saturated fatty acids	SFA						X	X
Solid proteins	SP							X

The abbreviations of the trait annotations are specified in the table.

In cattle, dairy QTLs are those reported in the studies of Sanchez et al*.* and Tribout et al*.*^26,28^. From daughter yield deviations, 84 QTLs were identified for milk production (milk, protein and fat yields), milk composition (protein and fat content) and mastitis resistance (clinical mastitis and somatic cell counts) in 6,321 Holstein, 2,515 Montbéliarde and 2,203 Normande bulls^28^. In addition, 34 QTLs were identified for milk protein composition (αs1, αs2, β and κ-caseins, β-lactoglobulin and α-lactalbumin) predicted from the mid-infrared spectra in 2,306 Holstein, 2967 Montbéliarde, and 2737 Normande cows^26^. A total of 1019 QTL data were listed for the three breeds and the different traits, corresponding to 118 distinct QTLs. In each study, confidence intervals of the QTLs were defined by the positions of the SNPs included in the upper third of the GWAS peak. As a result, the regions spanned a total length of almost 401 megabases, with a mean length of 815 kb (Table 5)*.*

In caprine studies, 82 QTLs for five different traits (fat content, protein content, fat yield, protein yield, and milk yield) were studied in two French alpine and Saanen dairy breeds^27^. Due to different reference genomes (Assembly CHIR_1.0 versus ARS1 (GenBank accession GCA_001704415.1)), the positions of the 82 QTLs were transposed to the ARS1 goat assembly. This step was carried out by selecting the 100 first and the 100 last nucleotides in each confidence interval of the QTLs using the Genome Data Viewer tool on NCBI^74^. Then, the flanking regions of each QTL were blasted via the online NCBI tool^75^. The NCBI megablast tool (“Highly similar sequences”) and the general parameters applied were those by default (short queries, expected threshold: 0.05)^75^. The QTL coordinates on the ARS1 genome version were deduced from the BLAST results. As a result, the 82 regions corresponded to a total length of almost 18 megabases, with a mean length of 226 kb (Table 5).

In ovine species, 107 dairy QTLs were available for Manech Tête Rousse and Lacaune breeds in the publication of Oget et al*.*^24^ and INRAE unpublished data (Hélène Larroque, personal communication). The QTL regions were transposed to the Oar_v4.0 genome version through the UCSC LiftOver tool^76^. Seventeen traits are represented in these dairy QTLs, listed in Table 2. These 107 regions corresponded to a total length of almost 77 megabases, with a mean length of 1197 kb.

##### miRNomes

To characterize the microRNAs expressed in the mammary gland and/or present in milk, dairy miRNomes were used. The miRNomes of bovine mammary glands with or without mastitis and the miRNomes of milk or different fractions of milk were used. A total of 1063 microRNAs are listed in these data sets^11,13–16^. For each listed microRNA, its genomic locations in the UMD3.1 bovine genome and its name were indicated.

In caprine species, the mammary gland miRNome data were available from Mobuchon et al*.*^12^. A total of 239 microRNAs are listed, and their genomic locations in the ARS1 genome version were used.

In ovine species, no mammary gland or milk miRNome data were available when the script was developed. Therefore, to prioritize ovine microRNAs detection, bovine and caprine miRNome data were used for the ovine species.

### Data filtering

**Figure 5:**
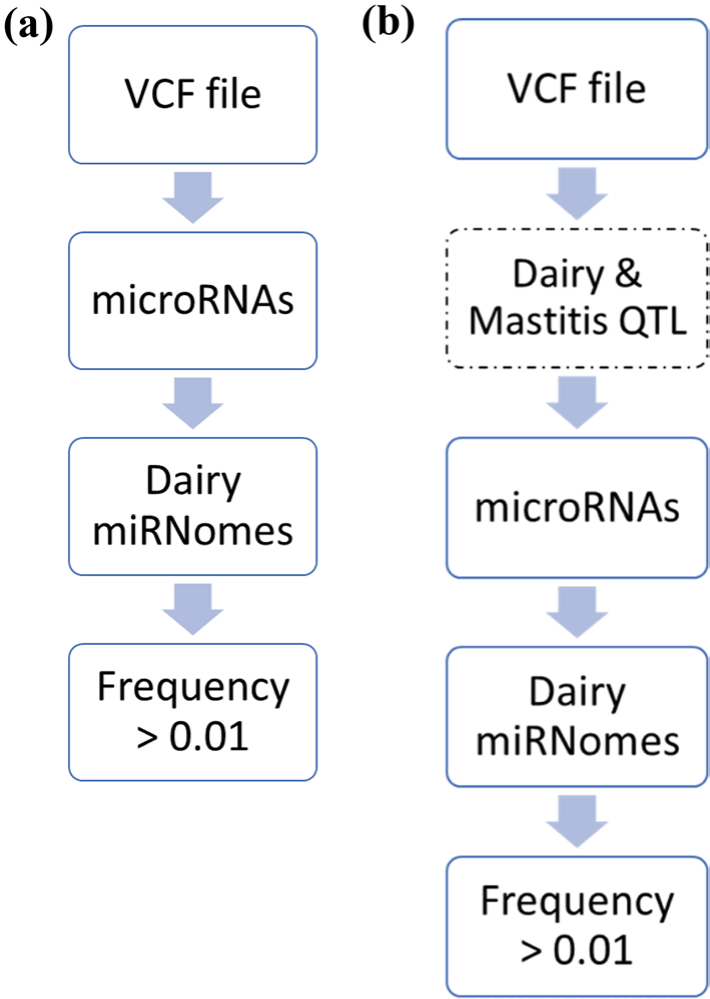
Data filtering process without (**a**) and with (**b**) the dairy and mastitis QTL filter. From the genetic variants in the VCF file, different filters were added to kept the variants of interest. The main steps of the pipeline are as follows and step 2 was present only in (**b**). (1) vcf files annotated with either VEP or SnpEff were used to filter only variants that are located in microRNA regions. (2) Positions of the previously filtered variants were used to select only those variants that are located within dairy QTL regions. (3) For each species, microRNA genes containing the filtered variants were subsequently compared to the species corresponding miRNomes of mammary gland or milk. (4) We selected variants with allelic frequencies of 1% or higher.

Files containing genomic variants, QTLs and miRNomes were combined and further analyzed using an in-house Python script. This script consisted of a cascade of several filtering steps allowing the selection of genetic variants located in dairy and/or mastitis QTLs, within microRNA gene regions expressed in the mammary gland and/or present in milk. The script used the microRNA, miRNome and frequency data as filters added in this script. First, only biallelic variants affecting microRNAs were further filtered from the annotated VCF files. Second, we selected genetic variants that impact microRNAs present in the dairy miRNome. Finally, the variants with a frequency higher than 0.01 were selected and corresponded to our miRNA genetic variant panel (Fig. 5a).

Then, the QTL data filter was added to this whole script to be more precise and more stringent. Only genetic variants located in dairy or mastitis QTLs were selected. Then, the microRNA, miRNome and frequency filters were processed (Fig. 5b). This panel also contained different features from the original three files, such as their genomic position, nature of reference, alternative alleles and their frequencies, the impacted microRNA, the breed in which the variant is polymorphic, and the QTL traits in which the variant was located. Additional filters were applied to the variants to classify them as follows: (i) variants located in microRNA precursors (in the seed, the mature microRNA out of the seed or in the precursor microRNA out of the mature) or (ii) in flanking regions of different sizes (50, 100, 500 or 1000 base pairs upstream or downstream of the microRNA precursor), which were predefined according to the literature data^77–82^, and (iii) in bovine species, CpG island or transcription factor binding site (TFBS) elements that could have an impact on gene expression were added to the scripts. These data were based on UCSC (https://genome.ucsc.edu) for the CpG islands and on Bickhart et al*.* for the TFBS data in bovine^83^.

Briefly, the main steps of the pipeline are as follows: First, vcf files annotated with either VEP or SnpEff were used to filter only variants that are located in microRNA regions. Second, positions of the previously filtered variants were used to select only those variants that are located within dairy QTL regions. For each species, microRNA genes containing the filtered variants were subsequently compared to the species corresponding miRNomes of mammary gland or milk. Finally, we selected variants with allelic frequencies of 1% or higher.

### Prediction of mRNA targeted by microRNAs

The TargetScan release 7.2 bioinformatic tool was used to determine putative mRNA targets of microRNAs of interest (http://www.targetscan/) in bovine, caprine and ovine species based on the bovine database. This tool predicts and lists mRNAs that are potential targets of each microRNA. The target prediction is based on the complementarity between the microRNA and mRNAs in the seed sequence corresponding to nucleotides 2 to 7^84^, which is the recognition site in the microRNA. Additionally, the transcripts from in silico prediction were compared to the transcriptome data of the bovine mammary gland during lactation and the dry period^43^.

## Supplementary Information

Supplementary Legends.

Supplementary Table S1.

Supplementary Table S2.

Supplementary Table S3.

Supplementary Table S4.
