# Supplementary material for: In silico identification of variations in microRNAs with a potential impact on dairy traits using whole ruminant genome SNP datasets
Source: Sci Rep. 2021 Oct 1;11:19580. doi: 10.1038/s41598-021-98639-9 (PMC8486775; doi:10.1038/s41598-021-98639-9)
Supplement: Supplementary file 1 — Supplementary Legends. [file 41598_2021_98639_MOESM1_ESM.docx]

"In silico identification of variations in microRNAs with a potential impact on dairy traits using whole ruminant genome SNP datasets

BOURDON Céline, BOUSSAHA Mekki, BARDOU Philippe, SANCHEZ Marie-Pierre, LE GUILLOU Sandrine, TRIBOUT Thierry, LARROQUE Hélène, BOICHARD Didier, RUPP Rachel, LE PROVOST Fabienne, TOSSER-KLOPP Gwenola"

Supplementary Table legends

Supplementary Table 1 (3 sheets): In the first column of each sheet is pasted the list of miRNAs presenting at least one variant in bovine (sheet 1), caprine (sheet 2) or ovine (sheet 3) species. In the second column, “0” indicates that the miRNA is not in a QTL region, wheras “1” indicates that the miRNA is localized in a QTL region.

Supplementary Table 2: Breed, Breed code and number of sequences used for SNP detection in cattle

Supplementary Table 3: Breed, Breed code, number of sequences, Continent, Country Code, country, for the 1159 goats used for SNP detection

Supplementary Table 4: Bioproject, biosample accession numbers and breed for the 86 sheep used for SNP detection
